# Effects of diffusional kurtosis imaging parameters on diffusion quantification

**DOI:** 10.1007/s12194-013-0206-5

**Published:** 2013-03-28

**Authors:** Issei Fukunaga, Masaaki Hori, Yoshitaka Masutani, Nozomi Hamasaki, Shuji Sato, Yuriko Suzuki, Fumitaka Kumagai, Masatsugu Kosuge, Haruyoshi Hoshito, Koji Kamagata, Keigo Shimoji, Atsushi Nakanishi, Shigeki Aoki, Atsushi Senoo

**Affiliations:** 1 Department of Radiological Sciences, Graduate School of Human Health Sciences, Tokyo Metropolitan University, 7-2-10 Higashiogu, Arakawa, Tokyo 116-8551 Japan; 2 Department of Radiology, Juntendo University, Tokyo, Japan; 3 Deparment of Radiological Medicine, The University of Tokyo Graduate School Medicine System Research Course, Tokyo, Japan; 4 Philips Electronics Japan, Ltd., Tokyo, Japan

**Keywords:** Magnetic resonance imaging, Diffusional kurtosis imaging, Diffusion tensor imaging, *b* value, MPG direction, Diffusion time

## Abstract

Diffusional kurtosis imaging (DKI) is a new technique based on non-Gaussian water diffusion analysis. However, the original DKI protocol (six *b* values and 30 motion-probing gradient (MPG) directions) requires more than 10 min of scanning time, which is too long for daily clinical use. We aimed to find suitable *b* value, MPG direction, and diffusion time settings for faster DKI. Four normal healthy subjects participated in the study. All DKI data sets were acquired on a clinical 3T-MRI scanner (Philips Medical Systems) with use of three protocols of 0–7500 s/mm^2^ *b* values, 6–32 MPG directions, and 23–80 ms diffusion time. There was a remarkable difference in the standard deviation (SD) of the mean DK values in the number of MPG directions. The mean DK values were significantly higher in the posterior limb of the internal capsule (*p* = 0.003, *r* = 0.924) and thalamus (*p* = 0.005, *r* = 0.903), whereas the mean DK values of the cerebrospinal fluid (CSF) (*p* = 0.001, *r* = −0.976) were significantly lower when we used a longer diffusion time. Our results indicate that the SD of the mean DK values was higher in 15 MPG directions than in 20 MPG directions and more. Because the mean DK values of the CSF were significantly lower when we used longer diffusion times, we expect longer diffusion times to be useful for DKI. We propose the following imaging parameters for clinical use: 0, 1000, and 2000 s/mm^2^ *b* values; 20 MPG directions; Δ/δ 45.3/13.3 ms.

## Introduction

In the technique known as diffusion-weighted imaging (DWI), the diffusion of water through biological tissue provides image contrast that depends on the Brownian motion of water molecules. This technique was introduced into clinical practice in the 1990s [1–3]. DWI can be used with echo planar imaging, through which it is possible to detect cerebral ischemia with imaging times ranging from a few seconds to 2 min [4]. Diffusion tensor imaging is a magnetic resonance imaging (MRI) technique enabling in vivo examination of white matter (WM) anisotropy in the human brain. Diffusion anisotropy is a parameter derived from the directional distribution of diffusivity, and the degrees of anisotropy have been shown to correlate with microstructural changes in neural tissues [5].

Diffusional kurtosis imaging (DKI) has been highlighted as a new technique based on non-Gaussian water diffusion analysis [5]. It is assumed that water diffusion in biological tissues is restricted. The non-Gaussian behavior of water molecules may provide useful information related to tissue structure and pathophysiology [2]. Many studies have been conducted with DKI for evaluation of cerebral infarction, glioma, multiple sclerosis (MS), Parkinson disease, attention-deficit hyperactive disorder, and others [6–11]. It is important to use DKI as a clinical tool for investigation of the imaging parameters in the healthy brain. In the DKI approach, it is desirable to acquire DKI datasets with multiple *b* value to minimize the fitting errors. However, the original protocol (six *b* values and 30 motion-probing gradient (MPG) directions) [5] requires more than 10 min of scanning time, which is regarded as being too long for daily clinical use. Moreover, to date, few reports have been conducted on the imaging parameters of DKI [12, 13] compared with those of diffusion tensor imaging and DWI [14–16].

Kurtosis is a statistical value showing the degree of deviation from a Gaussian distribution (kurtosis = 0). Parametric maps of the apparent kurtosis coefficient, $K_{\text{app}}$ (dimensionless), have been fitted to the following formula:

1 $$ S_{ \exp } = \left\{ {\eta^{2} + \left[ {S_{0} { \exp }\left( { - bD_{\text{app}} + \frac{1}{6}b^{2} D_{\text{app}}^{2} K_{\text{app}} } \right)} \right]^{2} } \right\}^{\frac{1}{2}} , $$

where $\eta$ is Rician noise and $D_{\text{app}}$ is the apparent diffusion coefficient (ADC) for a given direction [5].

The diffusion tensor has 3^2^ = 9 elements, but, because of symmetry, only 6 are independent. The diffusional kurtosis tensor has 3^4^ = 81 elements, but, because of symmetry, only 15 are independent. With these two tensors, $K_{\text{app}}$ in an arbitrary direction is calculated with the following formula:

2 $$ K_{\text{app}} = \frac{{{\text{MD}}^{2} }}{D_{\text{app}}^{2}}\sum\limits_{i = 1}^{3} \sum\limits_{j = 1}^{3}{\sum\limits_{k = 1}^{3} {\sum\limits_{l = 1}^{3} {n_{i} n_{j} n_{k} n_{l} W_{ijkl} } } } , $$

where MD is mean diffusivity and is the average of the diffusion coefficient overall directions, $n_{i} n_{j} n_{k} n_{l}$ denotes elements of the direction vector *n*, and *W* denotes elements of the diffusion kurtosis [12, 13].

Because the original DKI protocol (six *b* values and 30 MPG directions) takes too much time for daily clinical use, we sought DKI settings that result in faster scans. We examined the influence of various *b* values, MPG directions, and diffusion times on measurements of the mean DK.

## Materials and methods

Four normal healthy subjects (age range 21–24 years, mean age 22.5 years) participated in the study. This study was approved by the institutional review board of our hospital. Written informed consent was obtained from all participants and their relatives. All DKI data were acquired on a clinical 3T-MRI scanner (Philips Medical Systems, Best, The Netherlands) with use of three study protocols as follows:

Repetition time/echo time (TR/TE), 3000/99 ms; slice thickness, 5 mm; resolution, 2 × 2 mm; MPG directions, 32; *b* values, 0–7500 s/mm^2^ (16 steps, refer to Table 1). The time between the two leading edges of the diffusion gradient (Δ) and the gradient length (*δ*) were 49.1 and 39.1 ms. The total scan time was approximately 48 min 18 s.

**Table 1:** Protocol of *b* values and diffusional Kurtosis Imaging Metrics

Protocol No.^a^	Posterior limb of the internal capsule	Corpus callosum	Thalamus	Cerebrospinal fluid
FA				
1	0.75 ± 0.08	0.75 ± 0.06	0.33 ± 0.06	0.13 ± 0.04
2	0.74 ± 0.07	0.77 ± 0.05	0.32 ± 0.06	0.12 ± 0.04
3	0.73 ± 0.09	0.76 ± 0.05	0.29 ± 0.06	0.11 ± 0.04
4	0.70 ± 0.09	0.72 ± 0.05	0.30 ± 0.05	0.11 ± 0.05
5	0.66 ± 0.09	0.71 ± 0.03	0.24 ± 0.05	0.12 ± 0.05
ADC (×10^−3^ mm^2^/s)				
1	0.58 ± 0.03	0.84 ± 0.14	0.66 ± 0.14	1.90 ± 0.21
2	0.58 ± 0.04	0.78 ± 0.08	0.68 ± 0.18	1.88 ± 0.17
3	0.51 ± 0.02	0.71 ± 0.08	0.64 ± 0.17	1.64 ± 0.13
4	0.41 ± 0.02	0.56 ± 0.07	0.48 ± 0.07	1.11 ± 0.09
5	0.31 ± 0.02	0.38 ± 0.02	0.38 ± 0.03	0.66 ± 0.04
Mean DK				
1	1.22 ± 0.15	1.04 ± 0.13	0.91 ± 0.11	0.465 ± 0.068
2	1.02 ± 0.11	1.02 ± 0.09	0.71 ± 0.09	0.453 ± 0.055
3	0.89 ± 0.05	0.76 ± 0.05	0.62 ± 0.06	0.363 ± 0.028
4	0.85 ± 0.05	0.66 ± 0.05	0.63 ± 0.05	0.342 ± 0.025
5	0.72 ± 0.06	0.58 ± 0.05	0.56 ± 0.04	0.334 ± 0.024

Protocol 1: 0, 1000, and 2000

Protocol 2: 0, 500, 1000, 1500, 2000, and 2500

Protocol 3: 0, 500, 1000, 2000, 3000, and 5000

Protocol 4: 0, 1000, 3000, 5000, 6000, and 7000

Protocol 5: 0, 5500, 6000, 6500, 7000, and 7500

Uncertainties indicate standard deviation

^a^Each protocol number contains the following *b* values (s/mm^2^)

Protocol 1 used three *b* values, *b* = 0, 1000, and 2000 s/mm^2^, as proposed by Jensen et al. [13]. Protocol 2 used six *b* values, *b* = 0, 500, 1000, 1500, 2000, 2500 s/mm^2^, as the original protocol, which was proposed by Jensen et al. [5]. For Protocols 3–5, we prepared a combination that used six higher *b* values than those of Protocol 2, in order to compare these two protocols with mean DK values. Protocol 3 used *b* = 0, 500, 1000, 2000, 3000, and 5000 s/mm^2^. Protocol 4 used *b* = 0, 1000, 3000, 5000, 6000, and 7000 s/mm^2^. Protocol 5 used *b* = 0, 5500, 6000, 6500, 7000, and 7500 s/mm^2^.

TR/TE, 8000/90 ms; slice thickness, 3 mm; resolution, 3 × 3 mm; MPG directions, 6–32 (6 variations, refer to Table 2); *b* values, 0, 1000, 2000 s/mm^2^; Δ/*δ*, 44.1/34.5 ms. The total scan time was approximately 44 min 22 s. The scan time for 6 MPG directions was 2 min 26 s, for 15 MPG directions was 5 min 27 s, for 20 MPG directions was 7 min 7 s, for 24 MPG directions was 8 min 27 s, for 28 MPG directions was 9 min 48 s, and for 32 MPG directions was 11 min 7 s. To study the MPG direction, we evaluated the standard deviation (SD) of the mean DK value in WM and gray matter (GM).

**Table 2:** MPG directions and diffusional kurtosis imaging metrics

Motion probing gradient	Posterior limb of the internal capsule	Corpus callosum	Thalamus	Cerebrospinal fluid
FA				
6	0.65 ± 0.15	0.66 ± 0.14	0.37 ± 0.11	0.22 ± 0.12
15	0.63 ± 0.16	0.63 ± 0.13	0.35 ± 0.11	0.17 ± 0.11
20	0.61 ± 0.16	0.62 ± 0.15	0.29 ± 0.08	0.15 ± 0.11
24	0.59 ± 0.16	0.61 ± 0.17	0.29 ± 0.06	0.15 ± 0.11
28	0.60 ± 0.16	0.62 ± 0.16	0.28 ± 0.08	0.16 ± 0.12
32	0.63 ± 0.17	0.67 ± 0.15	0.29 ± 0.08	0.17 ± 0.13
ADC (×10^−3^ mm^2^/s)				
6	0.65 ± 0.06	1.02 ± 0.22	0.69 ± 0.09	2.24 ± 0.36
15	0.63 ± 0.05	1.02 ± 0.24	0.69 ± 0.08	2.22 ± 0.37
20	0.63 ± 0.05	0.97 ± 0.24	0.71 ± 0.08	2.22 ± 0.37
24	0.63 ± 0.06	0.99 ± 0.25	0.73 ± 0.11	2.20 ± 0.39
28	0.62 ± 0.05	0.98 ± 0.25	0.73 ± 0.13	2.08 ± 0.38
32	0.63 ± 0.05	0.99 ± 0.25	0.72 ± 0.14	2.07 ± 0.39
Mean DK				
6	1.21 ± 0.32	1.07 ± 0.31	0.94 ± 0.17	0.43 ± 0.24
15	1.26 ± 0.21	0.97 ± 0.26	0.99 ± 0.14	0.44 ± 0.34
20	1.25 ± 0.19	1.13 ± 0.26	0.90 ± 0.13	0.49 ± 0.16
24	1.21 ± 0.18	1.10 ± 0.31	0.94 ± 0.13	0.44 ± 0.17
28	1.28 ± 0.19	1.06 ± 0.28	0.97 ± 0.13	0.48 ± 0.20
32	1.28 ± 0.16	0.99 ± 0.24	0.97 ± 0.12	0.48 ± 0.18

Uncertainties indicate standard deviation

There was a negative correlation between the standard deviation of the mean DK and MPG directions in the posterior limb of the internal capsule and the thalamus

TR/TE, 8000/56–104 ms; slice thickness, 3 mm; resolution, 3 × 3 mm; MPG directions, 20; *b* values, 0, 1000, 2000 s/mm^2^; Δ/*δ*, 28.7–83.1/9.5–34.5 ms (6 variations, refer to Table 3). Total scan time was approximately 48 min 26 s.

**Table 3:** Diffusion time and diffusional kurtosis imaging metrics

Diffusion time^a^/TE (ms)	Posterior limb of the internal capsule	Corpus callosum	Thalamus	Cerebrospinal fluid	SNR
22.7/56	1.14 ± 0.17	1.21 ± 0.15	0.87 ± 0.11	0.47 ± 0.04	276 ± 48
40.9/70	1.21 ± 0.18	1.19 ± 0.23	0.88 ± 0.12	0.46 ± 0.05	208 ± 49
50.6/78	1.20 ± 0.21	1.22 ± 0.09	0.88 ± 0.11	0.44 ± 0.05	211 ± 54
62.2/88	1.23 ± 0.21	1.19 ± 0.15	0.92 ± 0.13	0.42 ± 0.06	162 ± 51
72.3/97	1.30 ± 0.20	1.09 ± 0.19	0.92 ± 0.14	0.41 ± 0.05	145 ± 67
79.9/104	1.37 ± 0.22	1.19 ± 0.12	0.96 ± 0.16	0.41 ± 0.05	135 ± 48

Uncertainties indicate standard deviation

^a^Diffusion time = Δ − *δ*/3 (ms)

Statistical analysis was performed with Scientific Package for Social Sciences, version 20 (SPSS, Chicago, IL, USA). We used Pearson correlation to investigate the relationships between mean DK and diffusion time, and the relationships between the signal-to-noise ratio (SNR) and diffusion time. We hypothesized that under ideal conditions, the cerebrospinal fluid (CSF) would have a Gaussian distribution. In other words, the mean DK value of the CSF would be close to zero. Therefore, we supposed that a lower mean DK value would improve the diffusion precision.

We calculated all diffusion metric maps such as FA, ADC, and mean DK, with the free software dTV.II.FZR (Image Computing and Analysis Laboratory, Department of Radiology, The University of Tokyo Hospital, Japan). The values of the DK tensor *W* and the diffusion tensor *D* can be obtained by means of the least squares method, which was proposed in [5, 12, 17]. Mean DK values were calculated from each DK value.

**Fig. 1:**
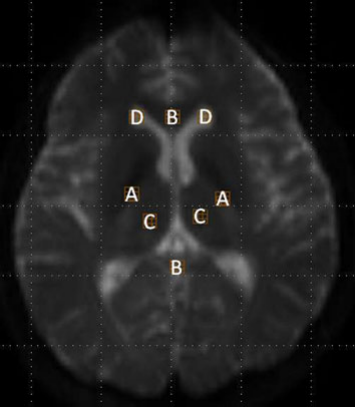
Superimposed volumes of interest on T2-weighted image without motion probing gradient. *A* Posterior limb of the internal capsule, *B* corpus callosum, *C* thalamus, *D* anterior horn of the lateral ventricle

The volumes of interest (VOIs) were placed on the posterior limb of the internal capsule (Fig. 1a), corpus callosum (Fig. 1b), thalamus (Fig. 1c), and anterior horn of the lateral ventricle (Fig. 1d).

To study diffusion time, we placed the region of interest (ROI) in the globus pallidus and the extra-cranial background region using MRIcro (free software), in order to measure the SNR. For DKI, the globus pallidus has been shown to be a useful region for the testing SNR at 3 T [13]. The SNR was defined as the mean signal intensity in the globus pallidus and the standard deviation of the noise in the extra-cranial background region [18]. In studying the VOI and ROI, we saved and used the same VOI or ROI for every subject and every protocol.

**Fig. 2:**
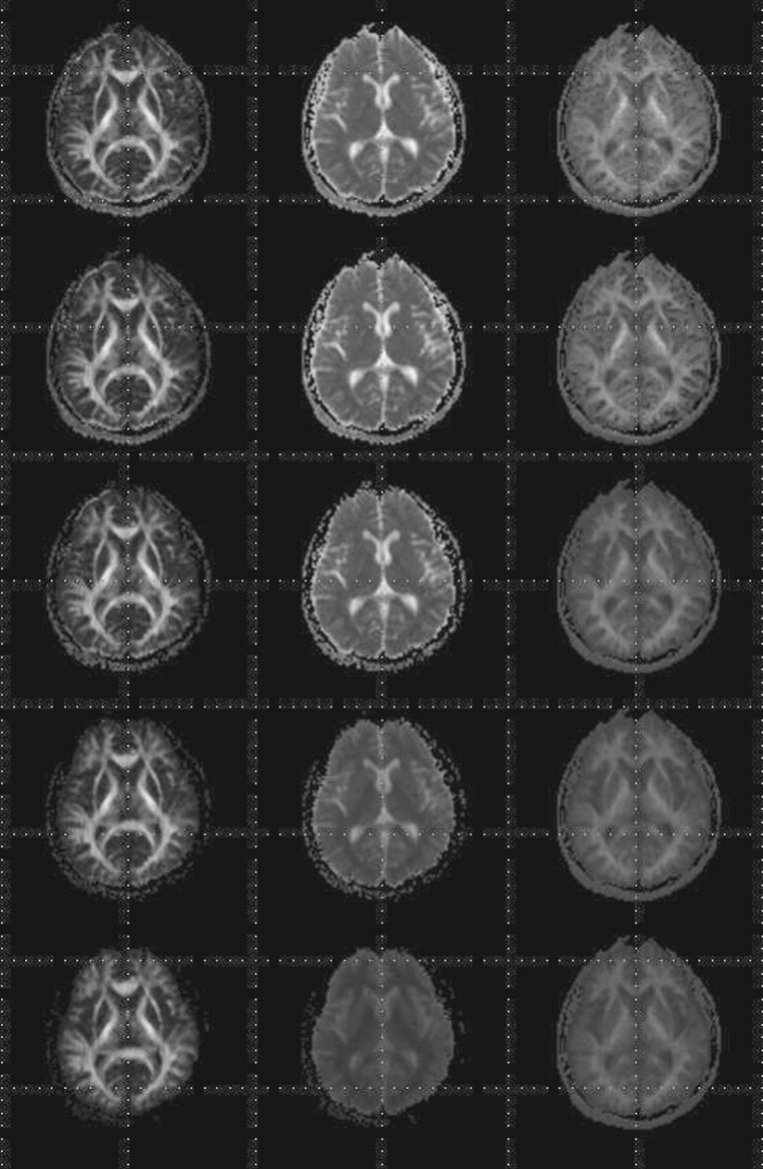
Left to right columns, fractional anisotropy (FA), apparent diffusion coefficient (ADC), and mean diffusional kurtosis (DK) maps of the brain of a healthy volunteer. *Top row* (Protocol 1): 0, 1000, and 2000 (s/mm^2^), *2nd row* (Protocol 2): 0, 500, 1000, 1500, 2000, and 2500 (s/mm^2^), *3rd row* (Protocol 3): 0, 500, 1000, 2000, 3000, and 5000 (s/mm^2^), *4th row* (Protocol 4): 0, 1000, 3000, 5000, 6000, and 7000 (s/mm^2^), *5th row* (Protocol 5): 0, 5500, 6000, 6500, 7000, and 7500 (s/mm^2^)

**Fig. 3:**
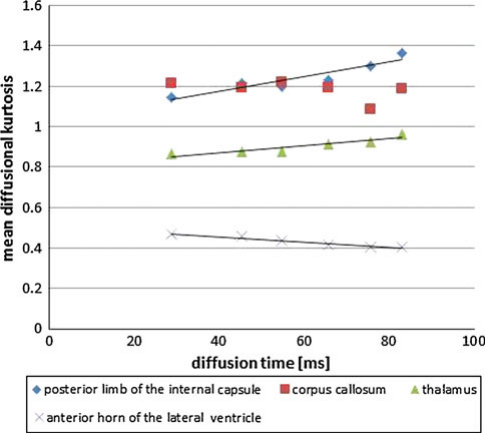
Relationship between mean diffusional kurtosis (DK) and diffusion time in the posterior limb of the internal capsule, corpus callosum, thalamus, and anterior horn of the lateral ventricle. The *solid lines* represent the linear regression line between mean DK and diffusion time

## Results

The fractional anisotropy (FA), ADC, and mean DK values were lower in the WM and GM with higher *b* values; this tendency was seen in the combination in which *b* values were above 6000 s/mm^2^ (Protocols 4 and 5) in the ADC (Table 1; Fig. 2).

The FA and ADC values did not differ in the number of MPG directions. However, there was a remarkable difference in the SD of the mean DK values (Table 2).

The mean DK values were significantly higher with use of a longer diffusion time in the posterior limb of the internal capsule (*p* = 0.003, *r* = 0.924) and thalamus (*p* = 0.005, *r* = 0.903), whereas the mean DK values for the CSF (*p* = 0.001, *r* = −0.976) were significantly lower with use of a longer diffusion time. The SNR decreased significantly with use of a longer diffusion time (*p* = 0.001, *r* = −0.978) (Table 3; Fig. 3).

## Discussion

Mulkern et al. [19] reported that the water signal decay of the human brain departs from the mono exponential behavior commonly assumed when ADC maps are generated in clinical practice, once the *b*-factor range is extended above 6000 s/mm^2^. Our results for the ADC maps were consistent with a previous study [19]; the poor contrast between WM and GM was seen in Protocols 4 and 5 (Fig. 2). From Eq. (2), it has been shown that the value of the apparent diffusion kurtosis coefficient can be influenced by the ADC [12].

Lu et al. [12] reported that one simulation using with use of typical parameters (ADC = 1 μm^2^/ms, apparent kurtosis coefficient = 1) showed that the quadratic approximation would no longer be valid when the *b* value was 3000 s/mm^2^ or larger. Therefore, the maximum *b* value should be limited to within about 3000 s/mm^2^ [12]. In our study for the mean DK maps, the poor contrast between WM and GM was seen in Protocols 3, 4, and 5 (Fig. 2).

It has been reported that the regional mean kurtosis values in the posterior limb of the internal capsule, in the body of the corpus callosum, and in the thalamus were 1.23 ± 0.09, 1.17 ± 0.07, and 0.86 ± 0.07, respectively [20]. Our results for Protocol 1 of the posterior limb of the internal capsule, corpus callosum, and thalamus were 1.22 ± 0.15, 1.04 ± 0.13, and 0.91 ± 0.11, respectively. Our results were consistent with a previous study [20] as regional mean kurtosis value in the posterior limb of the internal capsule, in the body of the corpus callosum, and in the thalamus. To acquire DKI data for the whole brain in a clinically acceptable time, we believe that three *b* values (*b* = 0, 1000, and 2000 s/mm^2^) may be useful.

It has previously been shown that for properly measuring the mean diffusional kurtosis, it is necessary to employ at least 15 different diffusion-encoding directions [5]. However, Latt et al. [21] reported that it might be sufficient to measure in only six diffusional directions in order to obtain a DK estimate, for example, in the assessment of MS lesions.

We focused on the SD of the mean DK value, and there was a remarkable difference in the SD of the mean DK values in the number of MPG directions. The difference in the SD of the mean DK values has influenced the signal loss or calculation errors due to MPG directions. Our results indicate that the SD of the mean DK values was higher in 15 MPG directions than in 20 MPG directions and more. The MPG directions should be therefore number 20 or more for evaluation of the mean DK value.

Jensen et al. [5] reported that the mean diffusional kurtosis value in freely diffusing water molecules is theoretically zero. However, Falangola et al. [22] reported that the histograms of the mean kurtosis values had peaks for CSF of approximately 0.45. Yang et al. [23] reported that pure CSF has an intrinsically low kurtosis due to flow effects.

We hypothesized that the mean DK value of the CSF would be close to zero, and our results indicate this hypothesis. Because the mean DK values were significantly lower when we used longer diffusion times, we expect longer diffusion times to be useful for DKI. However, diffusion in the CSF is not a Gaussian distribution, because of the flow effect, choroid plexus, and membranes.

There are some limitations to this study. First, the subjects in this study were small in number. Second, it is known that the kurtosis values are influenced by other factors, such as noise, motion, and imaging artifacts [13].

From the above results, we consider the following imaging parameters to be suitable for clinical use: MPG directions, 20; *b* values, 0, 1000, 2000 s/mm^2^; Δ/*δ* 45.3/13.3 ms.
